# Gerontological social workers’ perspectives about the future at the start of a COVID-19 vaccination program: A photovoice study

**DOI:** 10.1177/14680173221144412

**Published:** 2023-01-03

**Authors:** Sofia Fontoura Dias, Lia Araújo, Liliana Sousa

**Affiliations:** Department of Education and Psychology, University of Aveiro, Aveiro, Portugal; CINTESIS.UA – Centre for Research in Health Technologies and Services, University of Aveiro, Aveiro, Portugal; School of Education, Polytechic Institute of Viseu, Viseu, Portugal; CINTESIS-UP – Centre for Research in Health Technologies and Services, University of Porto, Viseu, Portugal; Department of Education and Psychology, University of Aveiro, Aveiro, Portugal; CINTESIS.UA – Centre for Research in Health Technologies and Services, University of Aveiro, Aveiro, Portugal

**Keywords:** Social work, aging, social work research, older people, intervention, group work

## Abstract

**Summary:**

The COVID-19 pandemic is a continuing public health crisis, although it has lessened in its intensity since the start of worldwide vaccination programs. In aged care facilities, gerontological social workers have become frontline professionals facing multiple challenges and demands. One year after the first COVID-19 case in Portugal, during the second major lockdown in the country, and with vaccination starting in these facilities, a photovoice program to identify the experiences of these professionals was developed. This study aimed to understand how gerontological social workers foresee the future of practice and intervention with older adults. A thematic analysis was conducted based on the photographs and associated narratives from 10 participants, all female, aged between 22 and 35 years, who attended a program’s session.

**Findings:**

Three themes were identified with the thematic analysis: (1) personal and professional growth (with renewed life perspectives and increased resilience), (2) reinvention of intervention (with improved management of emotions, teamwork, and alternative ways of intervening), and (3) hope to use the lessons learned (hope that vaccination will bring conditions to recover the older adults’ well-being and opportunities to use the good lessons learned).

**Applications:**

These findings are relevant to inform policymakers and governments about practices in aged care facilities and to improve the training of gerontological social workers in acute action management and intervention. We stress alternative ways of intervening that came up in the response to the pandemic such as emotional management, digital technology, communication strategies, self-care, or the families’ involvement.

## Introduction

On March 11, 2020, the World Health Organization declared the COVID-19 outbreak a global pandemic (Cucinotta & Vanelli, 2020). Protective measures were essentially based on social distancing, leading to consecutive lockdowns in homes and/or workplaces (Vanhaecht et al., 2020). Older adults, particularly those aged more than 80 years old, face a significantly higher risk of severe illness from the virus compared to the general population, caused by the physiological changes that develop with aging and underlying health conditions, as well as a higher risk of mortality (Kasar & Karaman, 2021; Kemenesi et al., 2020). Many older people live in aged care facilities, displaying high functional dependency, fragility, and chronic comorbidities (ECDC Public Health Emergency Team et al., 2020). Thus, aged care facilities constitute potential crowded hotspots for infection acquisition and spread, as the increased risk of contamination with the movement of visitors, staff, and users contributes to transmission routes (Kemenesi et al., 2020). Several prophylactic measures (e.g., from the Director-General of Health of Portugal) based on social distancing and isolation were developed and implemented to protect this age group and these collective facilities, which faced the emergent organization of a set of new and unknown procedures (Hashan et al., 2021). In addition, the institutions were instructed to restrict services (e.g., hairdressers), group activities, and communal dining, since these were considered “non-essential” activities. In some cases, activities outside the residents’ bedrooms were also constrained (El Haj et al., 2020).

This public health crisis required the mobilization of multidisciplinary teams of most frontline health professionals (Vanhaecht et al., 2020), who experienced high levels of burnout (White et al., 2021). In Portugal, most aged care facilities rely on gerontological social workers. However, their recognition (by the public and by researchers) as frontline professionals was less forthcoming, despite their efforts in the COVID-19 response in these facilities (Afonso et al., 2022). By October 1, 2021, 1862 articles with COVID-19 and “health professionals” OR “health workers” could be found on Web of Science. The same search with “social professionals” OR “social workers” displayed only 283 results. It is consensual that the pandemic still has a remarkable impact on social workers, with several psychosocial consequences (e.g., distress) and additional work overload (Miller & Grise-Owens, 2021). Despite this fact, the experiences of frontline social workers seem to still have been underreported.

In addition to professionals and aged care facilities, the COVID-19 pandemic challenged qualitative social and interprofessional research, leading to the creative adaptation of research methodologies (Santana et al., 2021; Sy et al., 2020). For instance, during the crisis, participants might be difficult to reach for several reasons: physical (such as social distancing protocols and shelter-in-place orders), psychological (mostly traumatic stress symptoms associated with the fear of getting infected and/or transmitting the virus to others), and ethical (particularly in respect for persons, beneficence, and justice) (Santana et al., 2021). Researchers required methods that would allow “direct” access and document meaningful life experiences, perspectives, and priorities (Santana et al., 2021). Some authors (Malka, 2021a, 2021b; Sy et al., 2020) suggested photovoice for remote data collection. Photovoice is participatory action research that combines participants’ photographs and voices (Rodrigues et al., 2008; Wang & Burris, 1997) to document life experiences, needs, difficulties, and desires in photographs that are discussed in critical reflection. The process allows for the catalysis of personal and community change (Rodrigues et al., 2008; Wang et al., 2000). It entails three major goals: (1) “to enable people to record and reflect their community's strengths and concerns,” (2) “to promote critical dialogue and knowledge about important community issues through large and small group discussion of photographs,” and (3) “to reach policymakers” (Wang & Burris, 1997, p. 370).

In September 2020, after some adaptation to the ongoing pandemic, we started to plan a photovoice program, “Eyes on the Pandemic.” It was undertaken with social workers in Portugal, the country in the European Union with the fourth most aged population (European Union, 2019), which was heavily affected by this pandemic (Shaaban et al., 2020). The group sessions were run from January to February 2021, around one year after the beginning of the COVID-19 pandemic, during the second major lockdown in the country, and with the vaccination program starting at aged care facilities. This study is based on a program session that aimed to understand how gerontological social workers foresee the future in terms of their practice with older people and how to be prepared for what a pandemic still involves in times of uncertainty regarding its development. The results are relevant to informing policymakers and governments about practice in aged care facilities and to improving the training of gerontological social workers, particularly in acute action management and intervention.

## Methods

The “Eyes on the Pandemic” program (conducted via Zoom videoconference software) aimed to document the lived experience of frontline gerontological social workers in aged care facilities. This program included seven sessions, each focused on a specific topic about the experience of working in an aged care facility during the pandemic (for more details: Araújo et al., 2021). The fourth session addressed the participants’ perspectives regarding the future of practice and intervention with older adults. This topic was considered by the participants and the research team to be particularly relevant since the vaccination program was starting and it was expected to be a transition moment. Participants were invited to present photographs and discuss the future in a still ongoing pandemic, with the following questions: *How will practice be with aged people in the future, how can we prepare for it, and what/who can help*? This topic was agreed 10 days before the group session with the participants (who are co-researchers, according to the photovoice method).

Participants were recruited from the researchers’ network and had to meet the following criteria: a degree in social work and working in an aged care facility since the beginning of the pandemic in Portugal. This session comprised 10 participants, all female, aged between 22 and 35 years (*M* = 28.4; *SD* = 6.6), and working in aged care facilities. Their roles in these facilities were manager/executive director (*n* = 4), developing counseling and leisure activities (*n* = 4), and being responsible for staff and quality procedures (*n* = 2). At the time of the program, their professional experience in aged care facilities was diverse, ranging from nine months to 12 years. One participant has a professional experience of 12 years, one participant of 10 years, one participant of six years, two participants of five years, one participant of four years, two participants with three years, one participant of one year, and one participant with nine months. Participants were informed about the objectives of the program and the study, its design and methods, and the required collaboration. In addition, ethical aspects (e.g., informed consent of the participants and those being photographed or authorized from institutions) were explained and required. The objectives of the study were congruent with the Helsinki Declaration and were approved by the Ethics Committee of the Polytechnic Institute of Viseu (No. 14/SUB/2020).

The program was facilitated by the second author (main facilitator) and supported by the first and third authors. Participants were invited to present and describe photographs taken with their technological devices (such as smartphones cameras, and computers) and to select a maximum of four photographs to facilitate the group discussion and enable all participants to share their perspectives in a 90-minute session. The photographs were presented by each participant to the group for critical reflection based on the PHOTO questioning technique (Hergenrather et al., 2009): “*Describe your **P**icture,” What is **H**appening in your picture?,” “Why did you take a photo **O**f this?,” “What does this picture **T**ell us about your life?,”* and *“How can this picture provide **O**pportunities for us to improve life?.”* Participants were free to complete their statements with other reflections considered relevant to give meaning and significance to the discussion.

The session was later transcribed and submitted to thematic analysis (Braun & Clarke, 2006) considering the photovoice’s principle of participants as experts on their experiences (Foster-Fishman et al., 2005). The data analysis involved the three authors, who were present in the photovoice session, and followed an inductive and bottom-up process. The first and second authors started by reading the transcripts and viewing the photographs to become familiar with the data. Then, they independently proceeded to organize the codes into themes, by a process of successive refinement. After, the first and second authors met to discuss the system of themes and discussed until agreement was reached. Then, the three authors met, to have another perspective on the themes. The discussion led to minor alterations in the name of the themes. Finally, and to assure that the analysis portrayed the participants perspectives, the themes (associated narratives and photos) were shared in a group session with the participants, who agreed to the analysis with minor suggestions on the narratives of each theme. Verbatim extracts were selected from each theme with the names changed to preserve the participants’ anonymity. A set of photographs that triggered the most dialogue among participants was selected to represent each theme. All participants authorized the publication of their photographs.

## Results

Three themes were identified with the data analysis: personal and professional growth, reinvention of intervention, and hope to use the lessons learned.

### Personal and professional growth

When considering the future, all participants referred to *personal and professional growth* grounded in the recognition of renewed life perspectives and increased resilience.

**Figure 1.**
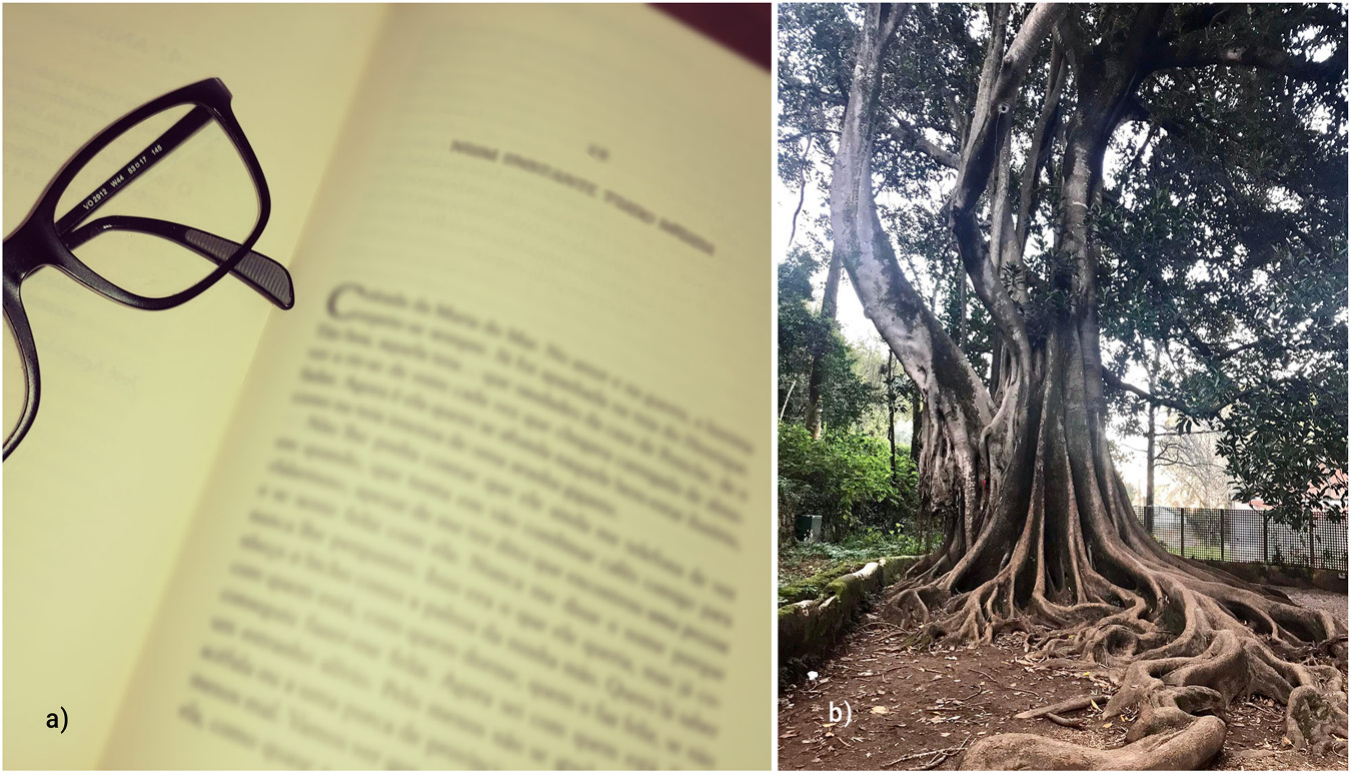
Sample photos of the participants on personal and professional growth. (a) “Being an open book” and (b) “The roots.”

The pandemic transformed life perspectives and personal values, since it revealed that uncertainty is present in our daily lives: “Do not make plans for life because life has plans for you” (Elsa, 34 years). Therefore, participants acknowledged that now it is vital “to give meaning to the simple things in life” (Esperança, 33 years). The future is now perceived as an *open book* (Figure 1a), where “I can write my own story differently due to the valorization of things that perhaps I did not value because they were taken for granted” (Ester, 25 years). This new understanding about life had repercussions on professional growth; thus, participants considered that they will be different professionals in the future. Their professional action is now even more guided by humanity values: “I consider myself human, but I think that I will be even more and more present” (Estela, 33 years) and overall, they “became better people” (Eva, 35 years).

Participants recognized that the pandemic had a profound impact in their lives, and that they will never be the same, but that it had also positive effects: “It left me a stronger person, more conscious, resilient. I think I found a strength in me that I did not know that I had” (Ema, 22 years). It was also stated that they should “come out of this a little stronger, as these roots” (Estefânia, 25 years; Figure 1b).

### Reinvention of intervention

Seven participants presented photographs on the *reinvention of intervention*, which included improved management of emotions, teamwork, and alternative ways of intervening. These professionals referred to enhanced management of emotions: “We have to know how to deal with our emotions, the emotions of the people we work with, and the emotions of those people's relatives and still try to convey a message of tranquility and serenity” (Elsa, 34 years). They had to support older people and their relatives in dealing with loneliness and emotional deprivation, due to the lack of touch, presence, and outside contact: “They have the need to hug their families […] and the families perhaps will come to visit more often” (Esperança, 33 years). In addition, the professionals had to deal with the fear and insecurity of older people and their relatives, as well as staff, regarding the virus transmission.

**Figure 2.**
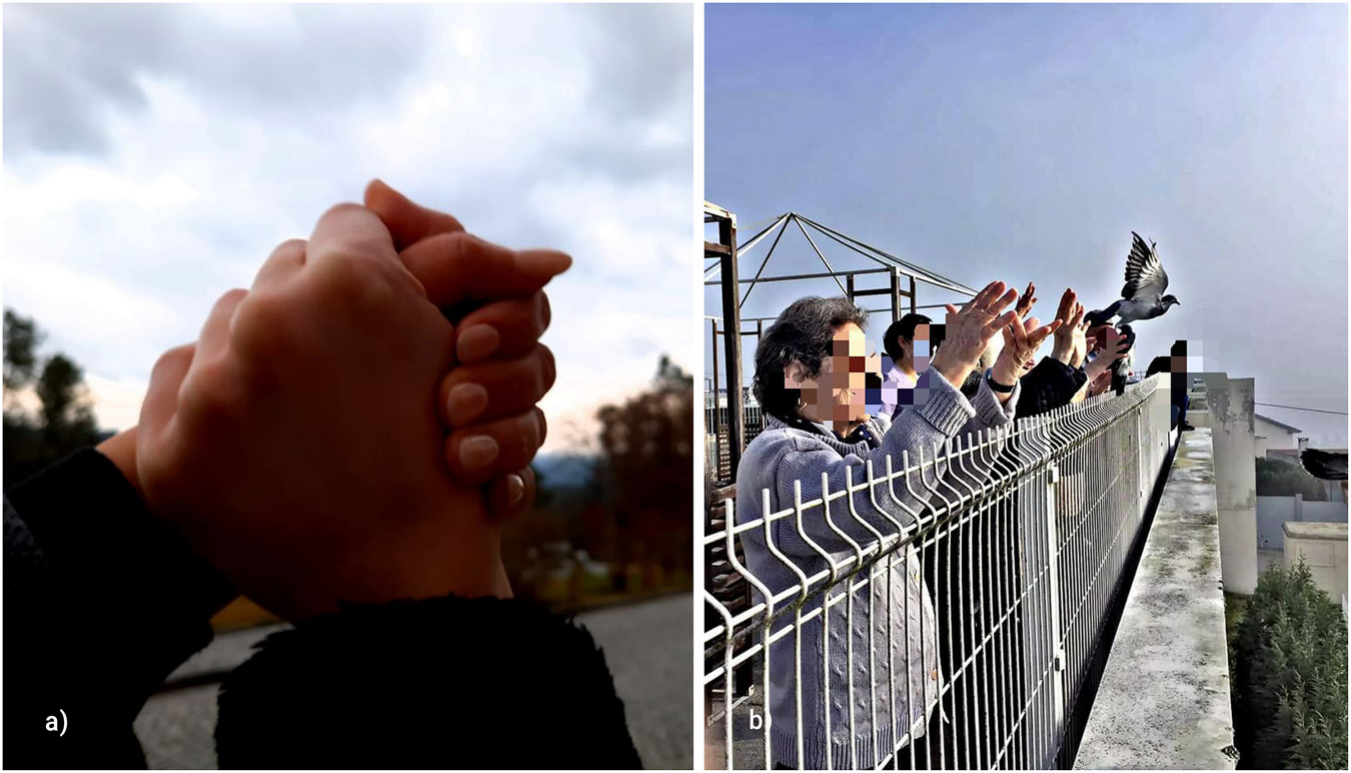
Sample photos of the participants on reinvention of the intervention. (a) “The only certainty in unity” and (b) “Peace.”

Participants reported that they relied more on “teamwork” when confronted with work overload (due to the prophylactic measures) and lack of staff (some were at home with their children or older relatives). The “strength of unity and teamwork” (Eliana, 25 years) among the staff was essential, which is pointed out as important for future practice: “Wherever I am in the future, whatever the context I am working in, I know that I will be a better professional because I learned how team unity can accomplish the impossible” (Eliana, 25 years). This idea is presented in Figure 2a.

Alternative ways of intervening emerged due to multiple outbreaks and new protective measures, especially prophylactic containment measures. Digital technologies were essential, and many digital barriers (e.g., lack of equipment and illiteracy) were overcome. Family visits were rapidly replaced by video calls, and activities turned to digital media, as expressed in this example: “At Christmas, we made a video […], and the families were more present […] a word, an image is enough for them […]” (Esperança, 33 years). Activities were organized for smaller groups or individuals and, whenever possible, carried out outside the institution, as represented in Figure 2b). Participants believed that these new ways of intervention ended up being good measures, which would remain in the near future to minimize the effects of the pandemic.

### Hope to use the lessons learned

All participants mentioned that they had hope that vaccination will bring the conditions to promote the recovery of the older adults’ well-being and to use what they have learned from this experience.

**Figure 3.**
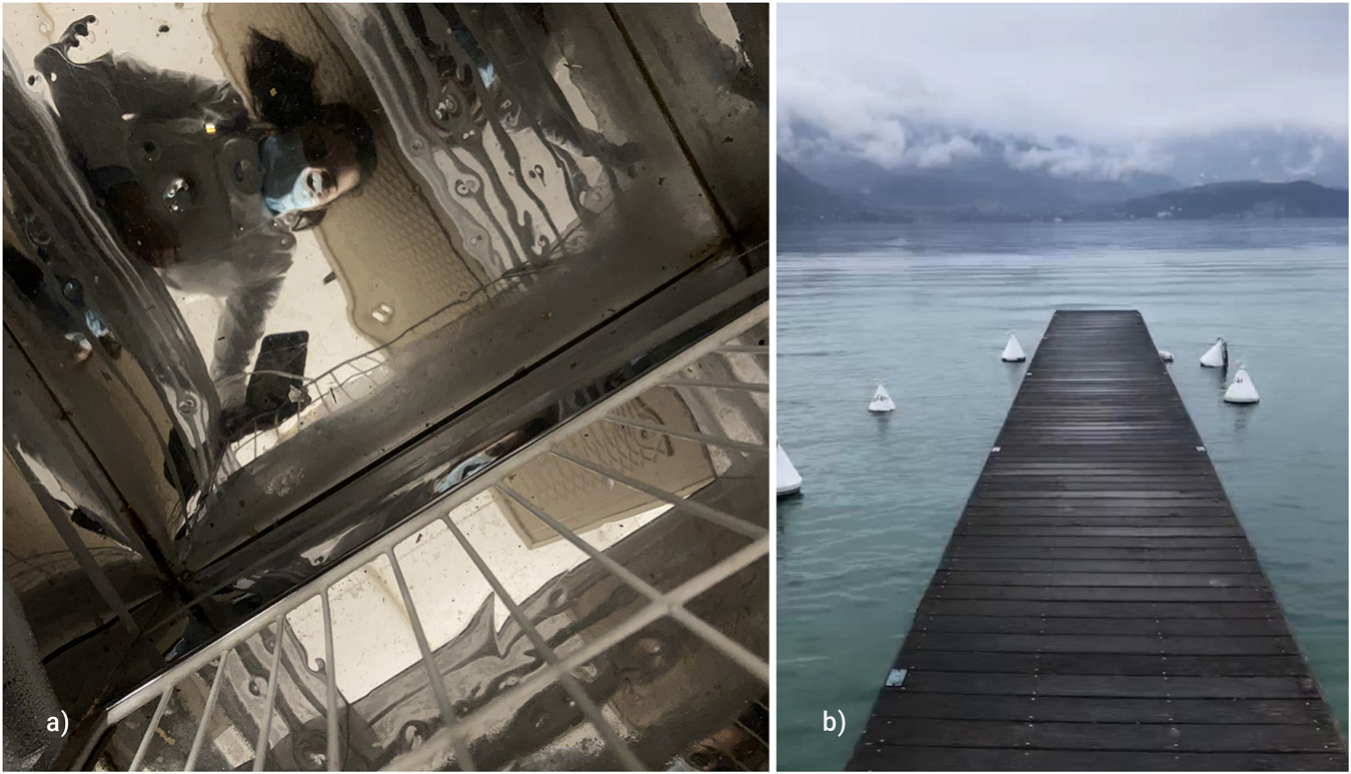
Sample photos of the participants on Eyes on the future. (a) “Light” and (b) “Breathe.”

The vaccination program started, and these professionals became hopeful: “We took today our second vaccine shot; therefore, we are starting to see a little light at the end of the tunnel” (Eva, 35 years; Figure 3a). However, uncertainties and doubts about the future were mentioned: “We are still not capable of thinking about the institution's future. The vaccination came to give a breath of fresh air and hope, but the future is still not that much thought of” (Ester, 25 years). As represented in one of the photographs presented in this theme, they already saw “a mountain on the other side of the river” (Ema, 22 years; Figure 3b). Participants underlined the importance of considering the impact of the pandemic on older adults’ well-being and health outcomes, since “we noticed a large mobility and basic functioning loss” (Ester, 25 years). In addition, they stated that “we still have a lot of work to do on the negative effects (loneliness and isolation) that this pandemic has brought to some of our users” (Eugénia, 28 years), whereby intervention practices will be focused on the mitigation of those harmful consequences with an even closer practice with the users, the valorization of outdoor activities, going outside to meet new places and activities involving families.

All the lived experiences and challenges led to important lessons learned that are opportunities to improve and that will be considered in future practice: “We are living today, [and will] learn from yesterday or try to do it” (Elsa, 34 years). The participants described the pandemic experience as an event “that is part of our history, it is a turning point” (Edite, 24 years). It was an opportunity for the professionals to reevaluate their actions and practices: “I perceive the future as an opportunity to improve the way we are working and learn from what, so far, may have not gone so well” (Estefânia, 28 years). This pandemic demonstrated the need to plan daily and adapt to what may come: “There is not one linear or straight way to guide us, but we face each situation as unique and take the best from it to surpass it” (Estefânia, 28 years). Participants stated that some practices started due to the pandemic will remain, such as the use of personal protective equipment by the staff: “We are so much more conscious of all risks, of the entrance of COVID and other diseases” (Ema, 22 years). They considered using digital resources in the future with older adults and their relatives, since the pandemic was considered a good opportunity for the families to reconnect with their loved ones.

## Discussion

At the start of the COVID-19 vaccination program in Portugal, this study used photovoice as a qualitative social research resource to identify, the perspectives of gerontological social workers regarding the future of their practice in aged care facilities. The thematic analysis demonstrated that participants highlight their (1) personal and professional growth, (2) reinvention of intervention developed, and (3) hope to use the lessons learned. When invited to reflect on the future, which followed their wish (since the topic was decided with the participants), participants started by getting a balance sheet of the recent pandemic past, highlighting their personal and professional growth during the pandemic. The gerontological social workers attached several difficulties as challenges from their lived experience and underlined the ongoing reinvention of their interventions. It is worth noting that this study's results must be understood considering when it was performed: around one year after the first COVID-19 case in Portugal, during the second lockdown, and with the vaccination starting in aged care facilities. During this initial period of acute crisis in the institutions, concerns were about outbreak control, regarding the safety of residents, relatives, and staff. The vaccination came as a “turning point,” which probably allowed the gerontological social workers to have a moment and opportunity to, for the first time, think about all they had gone through. Thus, with some tranquility came the positive realization that the past year had made them generally more resilient, strong, and confident. Due to this transformative event, the participants mentioned that they now had different values and life perspectives guiding their practice with aged people, such as humanization (“being even more human”), daily planning, and teamwork. This result goes in line with the international literature on social work responses and challenges during COVID-19, which accounts for the several ethical complexities professionals had to face. The pandemic was an opportunity to promote “creative responses, caring practice and pride at belonging to [this] profession” (Banks et al., 2020, p. 580).

Since the beginning of the COVID-19 pandemic, gerontological social workers have had to reinvent their interventions rapidly and effectively. The pandemic was related to changes in “social policies, welfare safety nets and the very foundations of the way we interact with each other” (Lewis, 2021, p. 52). For instance, Shrader et al. (2021) highlighted communication with families, the management of emotions (e.g., fear), and the effects of isolation on residents as the main challenges in long-term care facilities. In our study, the participants also identified the negative effects of social distancing on residents and staff and the importance of the involvement of families and relatives, highlighting how the intervention was reinvented to find solutions (e.g., family videoconferences). Digital resources came up as an ally for the effects of distancing, social isolation, and emotion management. In residential care facilities, where older adults are frailer, it is essential in the future to ensure staff's availability and preparation to assist users in the use of digital equipment and applications (Seifert et al., 2021). Teamwork and unity (of the staff, families, and residents) was another mentioned pillar of the intervention (Kar, 2020). As Kemp (2021) points out, when reflecting on “a new way forward” from this pandemic, “the dynamism and interdependence of care relationships, networks, and systems must be attended to and placed at the forefront of research, policy, and practice related to families and long-term care and quality improvement” (p. 149). In the future, all the mentioned alternative ways of intervening will be important topics of discussion since they were considered timely and positive changes.

In a world that is currently dealing with the negative consequences of acute critical interventions during the pandemic, professionals are now facing the daily recovery of older people in aged care facilities. This age group presents increased deficits in physical, cognitive, emotional, and social functioning (e.g., loss of mobility and basic cognitive function, and an increase in psychiatric conditions). Considering several biopsychosocial vulnerabilities of older people, social distancing is a cause of loneliness, which is a risk factor for depression and anxiety disorders (Banerjee, 2020; Kasar & Karaman, 2021). In addition, perhaps undervalued concerns have arisen with the physical and mental repercussions (for instance, burnout) on staff due to an increased workload, new roles and responsibilities, and uncertainty, fear, and emotional deprivation (El Haj et al., 2020; Kemp, 2021; White et al., 2021). Participants reported topics such as growth, reinvention, and hope; however, they were frontline professionals during very demanding times, and it became essential to make time and resources (e.g., self-care strategies and psychological support) available to them. In future, it is vital to improve the physical and mental recovery of professionals besides that of the residents.

The gerontological social workers reported that they perceive the future with hope, particularly due to the start of the vaccination program. They intend to use the good lessons learned to mitigate the negative repercussions of this pandemic on older people and to become even better professionals. Leotti (2021) described crises as opportunities to break with the past and envision a world anew. In this “new reality,” digital resources and the use of personal protection equipment were referred to by our participants as important tools. The gerontological social workers also added value to a professional practice based on the present with daily planning due to the unexpected. Within the next few years, it will be interesting to evaluate the maintenance of the activities, services, and ways of practicing that arose with the critical demands of this pandemic. Evidence from this follow-up photovoice session/program is part of a study regarding the pandemic evolution in aged care facilities.

The COVID-19 pandemic also impacted research activities. The “Eyes on the Pandemic” program was launched one year after the pandemic was declared, at the start of vaccination in aged care facilities, which was considered a turning point. We had wanted to start the program earlier to document the experience of gerontological social workers since the beginning of the pandemic; however, that was not possible. Nevertheless, the photovoice methodology allowed direct access to perspectives about the future from the lived experience of professionals concerning the first year of the COVID-19 pandemic. Photovoice emerged as a powerful tool that brought, from the *inside* to the *outside,* the lived experience of these professionals, keeping social distance and ensuring the safety of all involved.

As a methodology built upon critical reflection, it is plausible that more elaborate and positive reports from these professionals may emerge as verified. Participants were presented with a specific topic to photograph and then had about eight days to submit their photos, which could be taken in their personal or professional environments. This methodology allowed time to reflect on difficulties and increased resilience and strength.

## Limitations of the study

We are aware that our research has limitations. The first is that all reports are from qualified professionals. Aged care facilities comprise multidisciplinary teams that include other professionals (e.g., psychologists, nurses, gerontologists) and non-qualified staff (e.g., direct care workers). Further studies, along with follow-up sessions, should collect reports from other staff members to highlight co-existing different functions and perspectives. In addition, residents, families, and friends are other protagonists in the documentation of experiences in aged care facilities, whose reports may have become underreported as well.

## Conclusions

The COVID-19 pandemic was a “novel disease” (White et al., 2021, p. 851), since there was no adequate knowledge, training, preparation, or clear guidelines for professional practice. Frontline gerontological social workers performed critical roles as “buffers” against the lived uncertainty in this pandemic (Staller, 2021). Our results are relevant to inform policies and governments about intervention practices in aged care facilities. This historic event exposed the relevance of the professionals’ training in crisis management, particularly concerning emergency institutional responses, alternative ways of intervening based on emotional management, self-care, digital technology, communication strategies, and the families’ involvement. This study contributes to the documentation of the lived experience of gerontological social workers after one year of the COVID-19 pandemic and its impact on their perspectives about the future. This is also a report on the efforts of qualitative social researchers to remotely document the experiences of gerontological social workers from the *inside* to the *outside* in times of lockdown, safeguarding social distancing.
